# Gastrocnemius Recessions in the Management of Chronic Recalcitrant Plantar Fasciopathy—A Systematic Review

**DOI:** 10.3390/jfmk11010122

**Published:** 2026-03-17

**Authors:** Gianmarco Gemini, Antonio Mazzotti, Elena Artioli, Laura Langone, Federico Sgubbi, Alberto Arceri, Gianmarco Di Paola, Simone Ottavio Zielli, Cesare Faldini

**Affiliations:** 1 1st Orthopaedics and Traumatologic Clinic, IRCCS Istituto Ortopedico Rizzoli, 40136 Bologna, Italy; gianmarco.gemini@ior.it (G.G.); antonio.mazzotti@ior.it (A.M.); laura.langone@ior.it (L.L.); federico.sgubbi@ior.it (F.S.); alberto.arceri@ior.it (A.A.); gianmarco.dipaola@ior.it (G.D.P.); simoneottavio.zielli@ior.it (S.O.Z.); cesare.faldini@ior.it (C.F.); 2 Department of Biomedical and Neuromotor Sciences (DIBINEM), Alma Mater Studiorum University of Bologna, 40123 Bologna, Italy

**Keywords:** plantar fasciitis, plantar fasciopathy, gastrocnemius release, proximal medial gastrocnemius release, Barouk procedure, Baumann procedure, Strayer procedure

## Abstract

**Background**: Chronic Recalcitrant Plantar Fasciopathy (CRPF) is resistant to conservative treatments and has historically been managed with Open Plantar Fasciotomy (OPF). This systematic review aims to evaluate the role of the Gastrocnemius Release Procedures (GRPs) in treating CRPF, focusing on its indications, surgical techniques and clinical outcomes. **Methods**: A systematic literature search was conducted following PRISMA guidelines using MEDLINE, Cochrane and Scopus. Studies pertinent to the topic were screened, and those that reported clinical outcomes of GRPs in patients with CRPF were retrieved. The quality assessment was carried out using the Newcastle–Ottawa Scale. **Results**: Eighteen studies met the inclusion criteria, analyzing a total of 901 patients with a mean follow-up of 27.8 months. Indications for performing GRPs subsisted if conservative treatment failed to relieve pain and if Isolated Gastrocnemius Contracture (IGC) was present. All GRPs significantly reduced pain, with Visual Analogue Scale (VAS) scores decreasing from a mean of 7.3 pre-operatively to 2.56 post-operatively (64.93% reduction). American Orthopaedic Foot & Ankle Society (AOFAS) scores improved from 50.1 to 84.7 on average. Ankle dorsiflexion increased by an average of 7.75°. Patient satisfaction was high, with an average rate of 85% (range 61.6% to 100%). Minor complications were reported but resolved in most cases. **Conclusions**: Indications for performing GRPs still need to be clarified, and the best surgical technique remains to be defined. Nevertheless, the GRP seems to offer sustained pain relief and functional improvement in patients with CRPF.

## 1. Introduction

Plantar Fasciopathy (PF) is a degenerative process that involves micro-tears within the plantar fascia in a process called fasciosis. At the microscopic level, fascial thickening predominates over inflammatory changes [1]; hence the redefinition of the condition from plantar fasciitis to PF may better reflect the underlying pathology within the fascia [2]. In patients with moderate-to-severe persistent symptoms for at least 6 months, PF is considered chronic. If it remains unresponsive to conservative treatment, it is classified as recalcitrant, in which case surgery is recommended [3].

Chronic Recalcitrant Plantar Fasciopathy (CRPF) is a commonly reported cause of plantar heel pain and is usually seen as an overuse injury in athletes, runners and military personnel as well as in the general population [4]. Increased stress on the plantar fascia can be observed in obese patients [5,6], in individuals with work-related weight-bearing activities [7] and altered biomechanical conditions, such as flat foot [8] or cavus foot.

When treated, PF generally has a benign course, with resolution of symptoms in more than 80% of patients within 12 months [9]. Conservative therapy represents the first-line approach and involves various options. Anti-inflammatory drugs are frequently used. Corticosteroid, local anesthetic [10] and Platet Rich Plasma (PRP) injections [11] are also frequently used in clinical practice but with inconsistent results [10]. Physical therapy may include Transcutaneous Electrical Nerve Stimulation (TENS) [12], low-intensity laser therapy, heat therapy, Extracorporeal Shock Wave Therapy (ESWT) and soft tissue therapy. Stretching of the plantar fascia and Achilles tendon is considered one of the hallmark treatments for these patients [4]. Moreover, nocturnal dorsiflexion splints and orthotics [13] can be used.

When conservative treatment fails to relieve symptoms, surgery must be considered as an option. Until now, the gold standard for surgery for CRPF was Open Plantar Fasciotomy (OPF). The efficacy of this procedure was debated, mainly because of the high incidence of complications [14], including lateral column destabilization, medial arch collapse, Baxter’s nerve entrapment and neurovascular lesions [15], among others [16,17]. As such, new solutions to treat CRPF have been researched, and in the last few years Gastrocnemius Release Procedures (GRP) seems to be a promising solution [18].

The rationale of GRPs is rooted in the physiopathological mechanisms underlying CRPF. The persistent increase in strain on the plantar fascia caused by Isolated Gastrocnemius Contracture (IGC) leads to microtears and initiates fasciosis. The correlation between IGC and fascial strain was described by Dr. Amis J. as the “split-second effect”, and it occurs due to biomechanical alterations in gait. In a healthy gait cycle, during terminal midstance (the ankle rocker phase), a normally functioning gastrocnemius allows for sufficient dorsiflexion to dampen forces as the ankle moves through the end of its range of motion. However, with IGC, the second rocker phase is prematurely halted, causing an abrupt increase in strain on the plantar fascia. As a result, the forces acting on the fascia peak higher, faster and longer in time. Additionally, the knee plays a crucial role in this mechanism. When the second rocker is interrupted by the IGC, the knee is forced into terminal extension, creating an opposing force that further amplifies the strain in the plantar fascia. This cycle of excessive mechanical stress continues, progressively damaging the fascia until symptoms arise. The rationale of GR therefore lies in the restoration of adequate gastrocnemius length, allowing adequate force dampening at the end of terminal midstance [19].

Although GRPs are quite novel for the treatment of CRPF, their history dates back to the beginning of the last century, as they were used to treat equinus deformities of the foot in spastic or neurologically impaired patients. In 2002 Di Giovanni et al. described the association between IGC and foot pathology in non-spastic patients [20], expanding its indications.

This systematic analysis of the literature aims to evaluate the role of the GRP in the treatment of CRPF, with a focus on its indications, surgical techniques and clinical outcomes.

## 2. Materials and Methods

### 2.1. Search Strategy and Selection Criteria

This systematic review followed the Preferred Reporting Items for Systematic Reviews and Meta-Analyses (PRISMA) guidelines [21]. The MEDLINE (accessed via PubMed), the Cochrane Database of Systematic Reviews, and Scopus were systematically searched on 18 February 2026, for relevant publications on the use of GRPs in the treatment of CRPF. In addition, Google Scholar was used as a supplementary search tool to identify potentially relevant peer-reviewed articles not captured by the primary databases as a supplementary search tool to enhance search sensitivity. Two authors independently conducted the literature search. The results were filtered for studies published between January 2000 and January 2026. The PubMed (MEDLINE) search strategy was developed using a combination of Medical Subject Headings (MeSH) and free-text terms. The complete search string was: (Plantar Fasciitis [MeSH] OR Plantar Fascia [MeSH] OR plantar fasciitis [Title/Abstract] OR plantar fasciopathy [Title/Abstract] OR plantar heel pain [Title/Abstract] OR chronic heel pain [Title/Abstract]) AND (Gastrocnemius Muscle [MeSH] OR gastrocnemius recession [Title/Abstract] OR gastrocnemius release [Title/Abstract] OR proximal medial gastrocnemius release [Title/Abstract] OR PMGR [Title/Abstract] OR Strayer procedure [Title/Abstract] OR Baumann procedure [Title/Abstract] OR Barouk procedure [Title/Abstract] OR gastrosoleus recession [Title/Abstract] OR isolated gastrocnemius contracture [Title/Abstract]).

The results of the literature search were analyzed for relevant articles.

Only articles available in English were included. Each article needed to report data concerning the clinical outcomes of GRP; if accessories procedures or other pathologies were present, the study was included only if it was possible to clearly separate data pertinent to the PMGR and CRPF. Reviews, case reports, and studies that did not report clinical outcomes—such as surgical techniques or cadaveric studies—were excluded.

The titles and abstracts of the identified articles were independently analyzed and screened by two authors, and full-text versions of relevant articles were obtained for further evaluation. Any disagreements regarding the inclusion of articles were resolved through consultation with the senior author. Articles of interest were then selected for full-text analysis according to our inclusion and exclusion criteria.

### 2.2. Data Extraction and Reporting

The research question was defined according to the PICO framework as follows: patients with CRPF refractory to conservative treatment (Population), treated with gastrocnemius release procedures, including proximal medial gastrocnemius release and other gastrocnemius recession techniques (Intervention), compared with the preoperative baseline (Comparison), with outcomes assessed in terms of pain reduction, functional improvement, ankle dorsiflexion, patient satisfaction, and procedure-related complications (Outcomes).

The study characteristics retrieved included title, author, publication year, and level of evidence (LOE). Cohort characteristics retrieved included the number of patients and the total number of limbs undergoing GRP, the mean age at surgery, Body Mass Index (BMI), and the follow-up length. The surgical techniques used were recorded. Pre- and post-operative clinical scores and measurements were reported, including Visual Analog Scale (VAS) [22], American Orthopedic Foot & Ankle Society (AOFAS) score [23], Patient-Reported Outcomes Measurement Information System (PROMIS) [24], Short Form-36 (SF-36) questionnaire [25] and ankle dorsiflexion. The VAS is a subjective scale used to measure pain intensity and is scored from one to ten points. The AOFAS score is an outcome measure used for assessing ankle and hindfoot conditions that combine both patient-reported metrics such as pain and function and physician-reported metrics such as foot alignment. The PROMIS score is a system used to assess and monitor physical, mental, and social health in both the general population and individuals with chronic conditions. It is measured using a T-score metric, in which 50 is the mean of a relevant reference population and ten is the standard deviation (SD) of that population. For PROMIS measures, higher scores equal more of the concept being measured (e.g., more Fatigue, more Physical Function). This could be a desirable or undesirable outcome, depending upon the concept being measured. The SF-36 is a tool used to assess quality of life and the impact that a condition has on the life of an individual. It involves 36 questions and scores from zero to 100, where the lower the score, the more the disability. Complications were also recorded.

The selected studies were analyzed, and information of interest was extracted into a database created via Microsoft Excel for Microsoft 10 (Microsoft Corporation, Redmond, WA, USA). The extracted data were reported via descriptive statistics. Continuous variables are reported as the means and ranges (minimum–maximum). Categorical variables are expressed as frequencies and percentages.

### 2.3. Quality Assessment

The quality assessment was carried out via the Newcastle–Ottawa Scale (NOS) [26]; this is a tool used for assessing the quality of non-randomized studies included in a systematic review. Using the tool, each study is judged on eight items, categorized into three groups: the selection of the study groups; the comparability of the groups; and the ascertainment of either the exposure or outcome of interest for case–control or cohort studies respectively. Stars awarded for each quality item serve as a quick visual assessment. Randomized controlled trials were assessed using the Cochrane Risk of Bias 2 (RoB 2) tool. With this tool bias is assessed in five distinct domains. Within each domain, users of RoB 2 answer one or more signaling questions. These answers lead to judgments of “low risk of bias,” “some concerns,” or “high risk of bias”. A traffic light plot was then generated to clearly summarize the risk of bias across the analyzed studies.

## 3. Results

### 3.1. Search Results

The initial search yielded 1688 papers. Based on the title and abstract, 1436 studies were excluded. The remaining 252 papers were evaluated in detail to verify their congruence with the inclusion criteria; 37 papers were retrieved for full text screening. After full-text screening, a total of 18 studies met the inclusion criteria.

**Figure 1:**
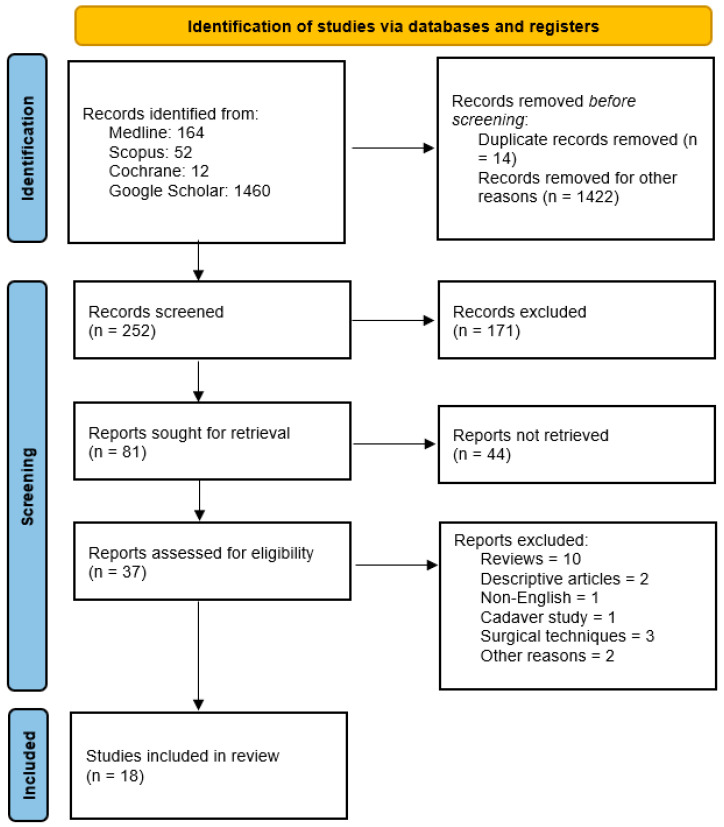
The PRISMA flow chart.

The PRISMA guidelines [21] were followed, and a flow chart was created to summarize the inclusion process of the analyzed articles (Figure 1).

### 3.2. Quality Assessment and Risk of Bias

The qualitative assessment for non-randomized studies was carried out via the NOS. Of the papers included, three were prospective studies, five were retrospective studies, and four were case series. Twelve studies scored between eight and ten points [18,27,28,29,30,31,32,33,34,35,36,37], only one study scored less than five points [29] (Table 1). For randomized study the RoB 2 tool was used. Three randomized studies were evaluated for quality, and all of them were assessed as being at a low risk of bias (Table 2).

### 3.3. Population

This study included 901 patients with a mean age of 46.31 years (range 18–70 years). A total of 923 limbs were treated surgically; bilateral procedures were performed in 22 patients (Table 3).

### 3.4. Indications

Once CRPF was diagnosticated, every patient was treated conservatively for 9.2 months on average (range 6–12 months) before undergoing surgery. In 17 out of 19 studies, the Silfverskiöld test was used to assess IGC to select patients who would benefit from GRP; in the other two studies, persistent pain after at least one year of conservative treatment was considered sufficient to indicate surgery.

### 3.5. Surgical Techniques

Various surgical techniques have been described involving different intervention levels. The recession level can be divided into three zones: zone one is defined from the gastrocnemius origin to the most distal fibers of the medial belly of the gastrocnemius; zone two is defined from the distal gastrocnemius belly to the end of the soleus muscle fibers; and zone three is defined from the end of the soleus to the distal insertion of the Achille’s tendon [30]. All the GRPs retrieved from the studies of this review were performed between zone one and zone two.

The following is a brief description of each of the techniques used in the papers:

- Proximal Medial Gastrocnemius Release (PMGR) procedure [29] is a zone one recession; the incision is made in the flexion crease of the knee, lateral to the medial fovea. The white fibers of the medial head of the proximal gastrocnemius aponeurosis are resected, and the ankle is dorsiflexed to check for recession.
- Baumann procedure [31] is a zone one recession; the incision is made at the mid-calf level along the medial aspect of the leg between the junction of the gastrocnemius and soleus muscle bellies. The crural fascia at the junction of the two muscles is divided, and blunt dissection is carried out from medial to lateral to create a space between them. Then, transverse incisions are made on the anterior aspect of the fascia. Finally, the ankle is dorsiflexed to check for recession.
- Strayer procedure [30] is a zone two recession; the incision starts two centimeters distal to the gastrocnemius indentation and extends proximally. The gastrocnemius tendon is incised distally at its insertion on the Achilles tendon, and the ankle is dorsiflexed to check for recession.
- Needle-based procedure [32] is a zone two recession performed using an Abbocath needle and ultrasound to guide the procedure. The needle is inserted medially in the space between the soleus and gastrocnemius muscles, then serum is injected to enhance visibility, and the needle is used to resect the white fibers while the ankle is dorsiflexed to assess recession and to allow for a clean cut with the needle.
- Endoscopic procedure [33] is a zone two recession; a small incision is made medial to the musculotendinous junction, a cannula is then inserted between the gastrocnemius and soleus muscles, and the fibers are carefully dissected through the cannula using a scalpel. The ankle is dorsiflexed to assess recession.

**Figure 2:**
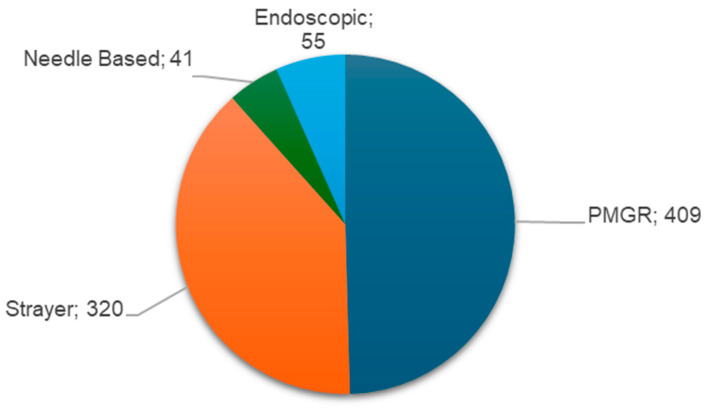
Surgical techniques and frequencies observed.

The PMGR was the most used GRP for a total of 409 cases, followed by the Strayer procedure for a total of 320 cases. A total of 41 patients were included in the needle-based study [34], and 55 patients underwent the endoscopic procedure [35] (Figure 2). Sanchez et al. [37,43] preferred the open Strayer method; however, if the myotendinous junction could not be adequately visualized the Baumann procedure was performed. Quantitative data regarding this procedure are lacking in the abovementioned papers.

### 3.6. Clinical Outcomes

All but two studies reported pre-operative and post-operative VAS scores. Overall, in all the studies, there was a considerable reduction in pain; across all the studies, the mean pre-operative VAS score was 7.3 (range 5.3–8.6), and the mean post-operative VAS score was 2.56 (range 1.2–3.3), indicating a 64.93% decrease in pain. The AOFAS score was the second most used tool to assess clinical outcomes; it improved from an average of 50.1 (range 39.1–65.3) pre-operatively to an average of 84.7 (range 78.8–93.3) post-operatively, showing an increase in function and a reduction in pain. Satisfaction was assessed via a Likert scale; in all the studies, most patients were satisfied, with an average satisfaction rate of 85% (ranging from 61.6% to 100%) (Table 4). The most pertinent domains of the SF-36 questionnaire were Bodily Pain (BP) and Physical Functioning (PF). The BP score increased from 31.9 (range 31–33) points on average pre-operatively to 48.6 (range 41–52.6) points on average post-operatively; the PF score increased from 56.9 (range 35.2–65) points on average pre-operatively to 63.8 (range 43.8–90) points on average post-operatively (Table 5). Ankle dorsiflexion was assessed as a metric of biomechanical function in 4 studies, reporting an average increase of 7.75° (range 5°–13°) in dorsiflexion post-operatively (Table 6).

The frequency of complications varies widely among studies. Upadhyay et al. [44] reported no complications in a series of 20 patients who underwent the Strayer procedure; Molund et al. [47] and Fike et al. [5] reported complications of up to 38% with the same procedure. No major complications were reported [48]; only minor complications were noted, mainly sural nerve lesions, in a total of 15 cases; wound complications were reported in a total of 7 cases; complex regional pain syndrome was reported in two cases; and thrombosis was reported in two cases among all 409 patients who underwent PMGR. In any case, most of those complications are resolved within a few months in most cases.

**Table 4:** Main clinical outcomes.

Reference		Cases	VAS Average		AOFAS Average		Likert Scale
			Preop	Postop	Preop	Postop	
Abbassian et al. [27]	2012	21	-	-	-	-	81%
Cheney et al. [38]	2018	73	7.4	2.8	-	-	-
Ficke et al. [5]	2018	18	8.3	2.4	-	-	-
Gamba et al. [46]	2020	15	6.81	3.63	65.3	87.1	85.8%
Hoefnagels et al. [39]	2021	32	7.8	24	-	-	78%
Huang et al. [40]	2018	10	6.8	2.4	39.1	78.8	75%
Iborra et al. [35]	2024	24	5.78	1.89	50.52	87.37	-
Koh et al. [36]	2022	55	7.2	1.7	42.6	86.2	80%
Maskill et al. [41]	2010	34	8	2	-	-	93.1%
Monteagudo et al. [18]	2013	30	8.2	1.8	46	85	90%
Molund et al. [47]	2018	40	7.6	3.3	59.5	88.0	-
Molund et al. [42]	2014	73	7	1.8	-	-	61.6%
Riiser et al. [45]	2024	21	7.6	2.8	59	88	-
Sanchez et al. [43]	2023	41	7.5	1.71	-	-	-
Sanchez et al. [37]	2023	189	5.53	2.11	-	-	-
Slullitel et al. [28]	2024	167	8.6	1.3	-	-	100%
Upadhyay et al. [44]	2021	20	7.2	1.2	49.4	93.3	90%
		Total:	Mean		Mean		Mean
		978	7.3	2.56	50.1	84.7	85%

**Table 5:** BP and PF domains of the SF-36 questionnaire.

Reference	SF-36 Items			
	BP Preop	BP Postop	PF Preop	PF Postop
Gamba et al. 2020 [46]	31.8	41.3	35.2	43.8
Huang et al. 2018 [40]	33.00	52.63	54.38	73.13
Koh et al. 2022 [36]	-	-	37.2	48.4
Molund et al. 2018 [47]	31	52	65	90
Mean	31.9	48.6	56.9	63.8

**Table 6:** Ankle dorsiflexion results.

Reference	Cases	Ankle Dorsiflexion	
		Preop	Postop
Hoefnagels et al. 2021 [39]	32	−5° (−20° to 2.5°)	10° (0° to 20°)
Iborra et al. 2024 [35]	24	0.89° ± 1.56°	18.79° ± 2.02°
Molund et al. 2018 [47]	40	6°	10.5°
Upadhyay et al. 2021 [44]	20	2.9°	16.35°

## 4. Discussion

In recent years, the understanding of PF physiopathology has advanced considerably: surgical indications have expanded, and GRP techniques have emerged as promising options. By addressing the underlying physiopathological mechanisms of PF—the increased strain on the plantar fascia due to IGC [19]—GRP has been shown to improve biomechanics. To appropriately select patients for surgery, a universally accepted definition of IGC and a standardized measurement method are necessary. Currently, the ST is the most used method to assess IGC; however, it lacks reproducibility and is operator-dependent. The use of goniometers for ankle range-of-motion assessment has been suggested as a more reliable alternative [49]. Some studies have demonstrated strong intrarater reliability for dorsiflexion measurements and moderate interrater reliability [45]. However, objective measurements should not be the sole determinant for surgery but should support clinical decision-making to provide strong evidence-based criteria.

With respect to surgical techniques, questions arise regarding the best procedure, both when comparing GRPs to OPF and when comparing different types of GRP. OPF has long been the gold standard in the treatment of CRPF, but in the late 1990s, new solutions were researched to assess the problem of high incidence of complications and low patient satisfaction [40,47]. As the GRPs spread, they proved to be effective and appreciated by both patients and surgeons. Until January 2025, studies that compare different GRPs are lacking, and the procedure of choice often depends on surgeon preference. Nevertheless, each GRP achieves the same goal, i.e., restoring normal gastrosoleus complex length, with all the benefits that come with it, including pain reduction and increased function. This statement is supported by the fact that all the studies reported a reduction in pain measured by the VAS and an increase in function measured by the AOFAS score. The longevity of these results was confirmed in four long-term studies [27,48,50,51]. It has also been reported that the combination of the GRP with other accessory procedures, including radiofrequency microtenotomy [39] or stretching [52], helps to both obtain and maintain good long-term results by preserving the optimal gastrocnemius length. In fact, an adequate length of the gastrocnemius is essential to provide those ten degrees of ankle dorsiflexion necessary for a physiological step scheme [53].

In this review, four authors reported the comparison between pre- and post-operative ankle dorsiflexion [35,37,40,51]; in particular, the mean pre-operative dorsiflexion angle was less than ten degrees, and the mean post-operative dorsiflexion angle was above it.

Finally, an important factor to consider is patient satisfaction. Ten studies evaluated satisfaction, reporting a mean of 85% (range 61–100%) satisfied patients. In the literature, OPF satisfaction rates vary widely; Davies et al. [50] reported a 73% satisfaction rate, Gibbons et al. [48] reported 72%, with 44% ongoing pain, swelling or tenderness, while Wheeler et al. reported 84% [54]. The main cause of patient dissatisfaction is complications. OPF is associated with plantar fascia rupture, wound complications, lateral column pain, and plantar nerve injury [46]. OPF can also cause biomechanical alterations such as loss of stability of the medial longitudinal arch and abnormalities in gait, particularly an excessively pronated foot [55]. Other patient complaints including long recovery times, as long as 7.8 months [40], contributed to the low satisfaction rates reported in many studies [47], and the following need to find an alternative procedure. On the other hand, GRPs are burdened mainly by minor complications that resolve within months in most cases. When comparing the satisfaction rates of different GRPs, each procedure seems to be appreciated by the patients, although the PMGR seems to be slightly better and has more consistent results across the studies included.

Hence, OPF and GR are both good options for treating CRPF with fewer long-term complications [18,42], making them viable and effective options.

Pickin et al. conducted a similar review in 2022 [55] and reached comparable findings. However, the present study advances this work by analyzing a larger number of articles, including some with extended follow-up periods—thereby providing stronger evidence that PMGR is a safe and reliable option. Nevertheless, the literature remains insufficient to designate PMGR as a new gold standard. More rigorous evidence is needed, particularly randomized controlled trials comparing different Achille’s lengthening procedures and GRPs to OPF, to better define treatment strategies and select interventions based on patient characteristics.

This study has several limitations. First, the included studies varied in methodology, follow-up duration, and outcome measures, which could introduce biases or affect the comparability of the results. In this review, the mean patient follow-up duration was 27.8 months; these data points included four outlier studies [28,29,48,50] that had long follow-up periods spanning from one to 14 years. In fact, if those outliers are excluded, the mean follow-up time decreases to 17.3 months (range 9–45). Furthermore, there is a lack of consensus on the indications and best surgical approaches. In this review, most patients were positive for the ST but not all of them. More studies should be conducted to understand whether IGC and ST are consistent metrics for selecting patients who could benefit from GRP.

Future developments in this technique will focus on minimally invasive surgery. Some authors have used endoscopic techniques [35,39] and needle-based eco-guided procedures [34] to minimize surgical trauma and speed up post-operative recovery, with promising results.

## 5. Conclusions

GRPs have emerged as a promising way to treat CRPF, particularly in patients presenting with IGC. Identifying IGC through a positive ST should be a common practice for patient selection, even though the exact criteria for surgical candidacy are still evolving. A variety of surgical techniques for treating GRPs have been described in the literature. Although PMGR seems to be the most widely used technique, there is no clear consensus on a superior method. Further high-quality randomized controlled trials are needed to clarify the optimal surgical indications, to compare GRPs directly with OPF, and to determine which GRP offers the most durable and reproducible results. The ongoing refinement and adoption of minimally invasive techniques may represent the next step forward in enhancing post-operative outcomes and patient recovery.

## Figures and Tables

**Table 1:** The Newcastle–Ottawa scale for assessing quality and risk of bias. List of abbreviations: CaD: Case Definition; Rep: Representativness; SNE: Selection of Non-Exposed cohort; DOI: Demonstration Outcome of Interest; AoE: Ascertainment of exposure; Com: Comparability; AoO: Assessment of outcome; FUL: Follow-up length; AFU: Adequacy of follow-up; Tot: Total.

Authors	Year	CaD	Rep	SNE	DOI	AoE	Com	AoO	FUL	AFU	Tot
Abbassian et al. [27]	2012	★	★	-	★	★	2 ★	★	★	★	9 ★
Cheney et al. [38]	2018	★	-	-	★	★	★	★	-	-	5 ★
Ficke et al. [5]	2018	★	-	-	★	★	★	★	★	★	7 ★
Hoefnagels et al. [39]	2021	★	★	★	★	★	★	★	-	★	8 ★
Huang et al. [40]	2018	★	★		★	★	★	★	★	★	8 ★
Iborra et al. [35]	2024	★	-	-	★	★	2 ★	★	-	★	7 ★
Koh et al. [36]	2022	★	★	★	★	★	2 ★	★	★	★	10 ★
Maskill et al. [41]	2010	★	★	-	★	★	★	★	★	★	8 ★
Monteagudo et al. [18]	2013	★	★	★	★	★	2 ★	★	-	★	9 ★
Molund et al. [42]	2014	★	★	-	★	★	★	★	★	★	8 ★
Shah et al. [29]	2022	-	★	-	★	★	-	-	★	-	4 ★
Sanchez et al. [43]	2023	★	★	-	★	★	★	★	★	★	8 ★
Sanchez et al. [37]	2023	-	★	-	★	★	★	-	★	★	6 ★
Slullitel et al. [28]	2024	★	★	-	★	★	★	★	★	★	8 ★
Upadhyay et al. [44]	2021	★	★	-	★	★	★	★	-	★	7 ★

**Table 2:** The RoB 2 traffic light plot. Green light means “low” risk of bias; yellow light means “some concenrns” about possible bias.

Reference	D1	D2	D3	D4	D5	Overall
Riiser et al. 2024 [45]	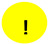	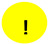		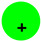	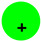	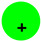
Gamba et al. 2020 [46]	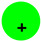	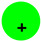	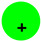	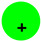	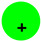	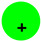
Molund et al. 2018 [47]	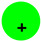	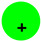	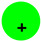	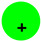	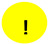	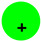

**Table 3:** Cohort characteristics.

Reference		Patients at Final Follow-Up	Limbs Treated	Mean Age	Mean BMI	Average Follow-Up (Months)
Abbassian et al. [27]	2012	17	21	52	-	24
Cheney et al. [38]	2018	68	73	-	-	-
Ficke et al. [5]	2018	17	18	46	34.7	20
Gamba et al. [46]	2020	15	15	46.2	31.7	12
Hoefnagels et al. [39]	2021	32	32	50	28.5	12
Huang et al. [40]	2018	8	10	45.5	26.4	12
Iborra et al. [35]	2024	19	24	41		9
Koh et al. [36]	2022	55	55	44.9	28.2	24
Maskill et al. [41]	2010	29	34	-	-	19.5
Monteagudo et al. [18]	2013	30	30	44	29.3	-
Molund et al. [47]	2018	40	40	46	27.8	12
Molund et al. [42]	2014	18	18	50		45
Riiser et al. [45]	2024	21	21	-	27.8	76.5
Shah et al. [29]	2022	115	115	-		6.6 y
Sanchez et al. [43]	2023	41	41	48	29	12
Sanchez et al. [37]	2023	189	189	40	30	51.96
Slullitel et al. [28]	2024	167	167	47	-	1–14 y
Upadhyay et al. [44]	2021	20	20	40.5	-	9
		Total:	Total:	Average:	Average:	Average:
		901	923	46.3	29.72	17.36

## Data Availability

No new data were created or analyzed in this study. Data sharing is not applicable to this article.

## Abbreviations

The following abbreviations are used in this manuscript:

CRPF	Chronic Recalcitrant Plantar Fasciopathy
OPF	Open Plantar Fasciotomy
GRP	Gastrocnemius Release Procedures
PRISMA	Preferred Reporting Items for Systematic reviews and Meta-Analyses
NOS	Newcastle-Ottawa Scale
IGC	Isolated Gastrocnemius Contracture
VAS	Visual Analogue Scale
AOFAS	American Orthopaedic Foot & Ankle Society
PF	Plantar Fasciopathy
PRP	Platet Rich Plasma
TENS	Transcutaneous Electrical Nerve Stimulation
ESWT	Extracorporeal Shock Wave Therapy
PROMIS	Patient-Reported Outcomes Measurement Information System
SF-36	Short Form-36
SD	Standard Deviation
BP	Bodily Pain
PF	Physical Functioning
